# The Administration of Hyaluronic Acid into the Temporomandibular Joints’ Cavities Increases the Mandible’s Mobility: A Systematic Review and Meta-Analysis

**DOI:** 10.3390/jcm11071901

**Published:** 2022-03-29

**Authors:** Maciej Chęciński, Maciej Sikora, Kamila Chęcińska, Zuzanna Nowak, Dariusz Chlubek

**Affiliations:** 1 Preventive Medicine Center, Komorowskiego 12, 30-106 Kraków, Poland; maciej@checinscy.pl; 2 Department of Maxillofacial Surgery, Hospital of the Ministry of Interior, Wojska Polskiego 51, 25-375 Kielce, Poland; sikora-maciej@wp.pl; 3 Department of Biochemistry and Medical Chemistry, Pomeranian Medical University, Powstańców Wielkopolskich 72, 70-111 Szczecin, Poland; 4 Department of Glass Technology and Amorphous Coatings, Faculty of Materials Science and Ceramics, AGH University of Science and Technology, Mickiewicza 30, 30-059 Kraków, Poland; kamila@checinscy.pl; 5 Department of Temporomandibular Disorders, Medical University of Silesia in Katowice, Traugutta 2, 41-800 Zabrze, Poland; zuzannaewanowak@gmail.com

**Keywords:** hyaluronic acid, temporomandibular joints, mandibular mobility, intra-articular administration

## Abstract

Objectives: The purpose of this systematic review with meta-analysis is to identify clinical studies concerning the impact of intra-articular administration of hyaluronic acid (HA) on mandibular mobility and to make an attempt at determining the efficacy of HA in this indication. Methods: The review included primary studies involving groups of at least 10 patients who were diagnosed with pain in the temporomandibular joint and who were injected with hyaluronic acid as the only intervention. The outcomes pursued were changes in mandibular mobility and pain intensity. Four databases of medical articles were searched, including PubMed and BASE. The risk of bias was assessed using the Cochrane methodology tools. The therapy‘s efficacy was calculated in the domains of mandibular abduction, protrusive movement, lateral mobility, and pain relief. For these values, the regression and correlation with variables characterizing the interventions were analyzed. Results: In total, 16 reports on 20 study groups with a total of 1007 patients qualified for the review. The mean effectiveness in the domain of mandibular abduction over the 6-month follow-up period was 122% of the initial value, and the linear regression model can be expressed as 0.5*x* + 36. The level of pain in the same time frame decreased to an average of 29%. The severity of pain 6 months after the beginning of treatment positively correlates with the number of injections per joint (0.63), the total amount of drug administered in milliliters (0.62), and the volume of drug administered monthly per joint (0.50). Limitations: In some studies, the patient groups were heterogeneous in terms of diagnosis. The studies varied depending on the joint into which the HA was administered. The synthesized studies differed with regard to the method of measuring the mandible abduction amplitude. Conclusions: The increase in the amplitude of mandibular abduction was expressed as the quotient of the mean values during the observation periods, and the initial value was achieved in all study groups, and in the linear regression model, it was 0.5 mm on average per month. Multiple administrations of the drug may reduce the analgesic effectiveness of the treatment.

## 1. Introduction

### 1.1. Rationale

The mandible’s movement is caused by the contraction of numerous muscles that operate the paired temporomandibular joints (TMJs). Restrictions in the mobility of the mandible can be a consequence of TMJ pain, classified in accordance with, among others, the International Classification of Orofacial Pain, 1st edition (ICOP) [1,2]. There is a substantial number of diverse treatments for abnormal function and hence pain in these joints [3,4]. The most frequently used methods of treating TMJs dysfunction include pharmacotherapy, physiotherapy, splint therapy, surgery (arthroscopy in particular), and intra-articular punctures [3,4]. The latter may be rinsing of the joint cavity (called arthrocentesis), intra-articular administration of autogenous blood products (e.g., platelet-rich plasma (PRP), or injectable platelet rich fibrin (I-PRF)) or drugs (e.g., corticosteroids) [3,4,5,6,7]. In addition, TMJ punctures allow for viscosupplementation, i.e., administration of the synovial fluid’s main component, hyaluronic acid (HA) [3,4].

The administration of HA into the TMJ cavity is a surgical procedure that does not require hospitalization, same as arthroscopy; multiple synovial fluid replacements, e.g., arthrocentesis; additional puncture into a peripheral vein, such as therapy with blood products; or administration of drugs with many known side effects, for instance, intra-articular corticosteroid therapy [3]. The absence of the above-mentioned drawbacks and numerous positive evaluations of HA administration to TMJ suggest that this therapy may be the solution of choice in many circumstances, especially in patients for whom a more invasive treatment is contraindicated.

### 1.2. Objectives

The purpose of this systematic review with meta-analysis is to identify clinical studies on the impact of intra-articular administration of HA on mandibular mobility and make an attempt to determine the efficacy of HA in this indication.

## 2. Methods

### 2.1. Eligibility Criteria

The inclusion and exclusion criteria of studies from the review were determined in accordance with the PICOS methodology, the name of which is an acronym for the following (Table 1) [8]. For each type of criteria, the inclusion was of paramount importance, and only after its fulfillment was the exclusion criterion verified. No limit was applied with regard to the publication time of the reviewed articles.

### 2.2. Information Sources

The selection of search engines was made in accordance with the recommendations of Gusenbauer et al. of 2020 regarding the selection of search systems for the purposes of systematic reviews and meta-analyses [9]. Of the 14 recommended by these authors as primary sources of information, all 4 search engines with open access and complex query capability were used. The following record providers were therefore decided on: (1) Association for Computing Machinery (ACM; over 3,000,000 records); (2) Bielefeld Academic Search Engine (BASE; over 278,000,000 records); (3) U.S. National Library of Medicine: ClinicalTrials.gov (about 400,000 records); (4) U.S. National Library of Medicine: PubMed (over 33,000,000 records) [10,11,12,13].

### 2.3. Search Strategy

The queries resulting from the previously determined eligibility criteria were expressed in the form of appropriate strings, slightly different for each search engine (Table 2). All databases were searched on 27 December 2021.

### 2.4. Selection Process

The selection of studies was made in accordance with the Preferred Reporting Items for Systematic reviews and Meta-Analyzes (PRISMA) methodology [14]. PRISMA 2020 Checklist and PRISMA 2020 for Abstracts checklist are Supplementaries S1 and S2, respectively [14]. A search of the databases yielded records containing the authors, title, year of publication, and journal in which each of the papers was published. These records were entered into the Rayyan tool (Qatar Computing Research Institute, Doha, Qatar and Rayyan Systems, Cambridge, MA, USA), which was used for blind abstract screening by two of the authors of this systematic review (M.C and K.C.) [15]. These authors made decisions as to whether to reject or further process the records. The convergence of these assessments was expressed by the Cohen’s kappa coefficient (*κ*), according to the formula:

*κ* = (*p*_0_ − *p_e_*)/(1 − *p_e_*),

where *p*_0_ is a relative observed agreement among raters and *p_e_* is a hypothetical probability of chance agreement [16].

In case of contradictory opinions, the given paper was processed further. The full-text evaluation was prepared by the same authors, and in the event of a possible discrepancy in decisions at this stage, the final judge was to be the next author (M.S.), but this was not the case during the selection process.

### 2.5. Data Collection Process

All data coming from the reports were then extracted without the use of automation tools. Author M.C. and an independent individual not belonging to the authors’ group (see: Acknowledgements) collected the data autonomously. Another author (K.C.) verified the correctness of the extracted data.

### 2.6. Data Items

The following data were extracted from the reports:

(1) First author of the report; (2) year of publication of the report; (3) designation of the study group; (4) type of administered HA; (5) amount of HA administered, ml per injection; (6) number of HA applications per joint; (7) total amount of HA administered, ml per joint; (8) total duration of therapy, weeks; (9) mean HA treatment interval, weeks; (10) mean amount of HA administered monthly, ml per joint; (11) other interventions in the study group; (12) study group size; (13) diagnosis according to ICOP; (14) number of joints treated; (15) number of right joints treated; (16) number of left joints treated; (17) mean number of joints treated per patient; (18) mean initial pain value on the Visual Analogue Scale (VAS); (19–25) mean VAS pain value at 1, 2, 3, 4, 5, 6, and 12 months; (26) mean initial mouth opening without pain; (27–33) mean pain free mouth opening after 1, 2, 3, 4, 5, 6, and 12 months; (34) mean initial unassisted mouth opening; (35–41) mean unassisted mouth opening after 1, 2, 3, 4, 5, 6, and 12 months; (42) mean initial assisted mouth opening; (43–49) mean assisted mouth opening at 1, 2, 3, 4, 5, 6, and 12 months; (50) mean initial protrusion movement; (51–57) mean protrusive movement after 1, 2, 3, 4, 5, 6, and 12 months; (58) mean initial painful joint side movement; (59–65) mean painful joint side movement after 1, 2, 3, 4, 5, 6, and 12 months; (66) mean initial movement to the healthy side; (67–73) mean healthy side movement after 1, 2, 3, 4, 5, 6, and 12 months; (74) mean initial movement to the right; (75–81) mean right movement after 1, 2, 3, 4, 5, 6, and 12 months; (82) mean initial movement to the left; (83–89) mean left movement after 1, 2, 3, 4, 5, 6, and 12 months.

Variables numbered from 1 to 17 characterize the study groups. Variables from 26 to 49 correspond to the primary outcomes set out in the PICOS criteria of this systematic review. The values of the variables 18–25 and 50–89 constitute secondary outcomes. In the absence of data for the desired observation period, data for the closest period of time were entered, if available. In the absence of other data, the fields of the summary table were left blank, and individual barrage values were not taken into account in the course of the meta-analysis.

### 2.7. Study Risk of Bias Assessment

The risk of bias in randomized controlled trials was assessed using “A revised Cochrane risk-of-bias tool for randomized trials” (RoB 2) [17]. In non-randomized trials, the Cochrane tool “Risk of Bias in Non-Randomized Studies of Interventions” (ROBINS-I) was intended to be used [18]. According to the instructions, the use of ROBINS-I was possible only for studies with at least 2 groups of patients [18]. The remaining studies were considered to be at high risk of bias. RoB 2 and ROBINS-I tools also served to assess the risk of bias due to missing evidence. The risk of bias was assessed independently by two authors of this systematic review (M.C. and Z.N.). All studies qualified for systematic review were included in the quantitative analysis, regardless of the possibilities and results of the risk assessment of bias.

### 2.8. Effect Measures

For the change in pain intensity and all indicators of mandibular mobility, the coefficient of effectiveness (*e*) was calculated according to the formula:

*e* = (*v_x_*/*v*_0_ − 1) × 100%,

where *v*_0_ is the baseline value and *v_x_* is the value *x* months after the therapy initiation.

### 2.9. Synthesis Methods

#### 2.9.1. Efficiency Evaluation

The condition required to include a given group of HA treated in the collective synthesis of effectiveness was the ability to calculate at least one value of effectiveness in a given domain. The quantitative synthesis covered only the groups treated with HA and was carried out in the domains: (1) maximal mandibular abduction; (2) range of mandibular protrusive movement; (3) the range of lateral mobility of the mandible; (4) joint pain. In the above-mentioned domains, the relative coefficients of the opening efficiency, protrusion, and lateral mobility, as well as changes in the level of pain, were taken into account, independent of the absolute values of individual variables. In the case of several measurement methods in (1) domain, the maximum unassisted opening of the mouth was included in the synthesis, and in its absence, the maximum abduction of the mandible without pain. The maximum assisted opening was only taken into account in the absence of both of the above-mentioned measurements. Due to the assessment of the lateral mobility range of the mandible in some studies, taking into account the healthy and the diseased side, and in others the right and left side of the body, the arithmetic mean of the effectiveness for lateral movements was calculated according to the formula:

*e* = (*e*_1_ + *e*_2_)/2,

where *e*_1_ and *e*_2_ are different efficiencies of lateral mobility within one test group.

#### 2.9.2. Regression Analysis

Meta-regression analysis was performed for each of the domains. The fitted regression models are shown as trend lines in graphs showing the change in treatment effectiveness over time. This analysis, apart from determining the averaged effectiveness of therapy with intra-articular injections of HA, allowed for the indication and an attempt to interpret the outliers. The coefficient of determination was denoted as *R*^2^.

#### 2.9.3. Correlation Analysis

The correlation was searched between data items (5)–(10), (12), (14), (17), the initial values of variables, and efficiencies in domains (1)–(4) after 1 and 6 months. In case of the unknown value of the variable after 6 months, the value after 12 months was given for the purposes of this analysis, and in the absence of both, the closest. Coefficients with more than half of the missing data were discarded. To determine the correlation, Pearson’s correlation coefficients (*r*) were calculated and presented in the form of a matrix. To calculate the t-score (*t*) of each correlation, the following formula was used:

*t* = *r*√(*n* − 2)/√(1 − *r*^2^),

where *n* is a sample size. Test probability (*p*) was calculated using the two-tailed Student distribution. The adopted significance level was α = 0.05.

### 2.10. Researchers’ Experience

The team of authors of this article prepared to conduct the described systematic review by taking introductory lessons in the field of conducting reviews (Jagiellonian University, Krakow, Poland), studying the relevant guidelines, carrying out exercises in the form of pilot reviews, and conducting other systematic reviews [19,20,21,22,23,24,25,26]. The experience of M.C. and M.S. in developing eligibility criteria and search strategies is supported by six systematic reviews published so far [20,21,23,24,25,26]. Authors M.C. and Z.N. assessed the risk of bias to date in two published systematic reviews [23,25]. Led by M.C., K.C., and Z.N., data extraction as a stage of scientific work resulted in the publication of eight reviews, six of which were systematic [19,20,21,22,23,24,25,26].

## 3. Results

### 3.1. Study Selection

**Figure 1:**
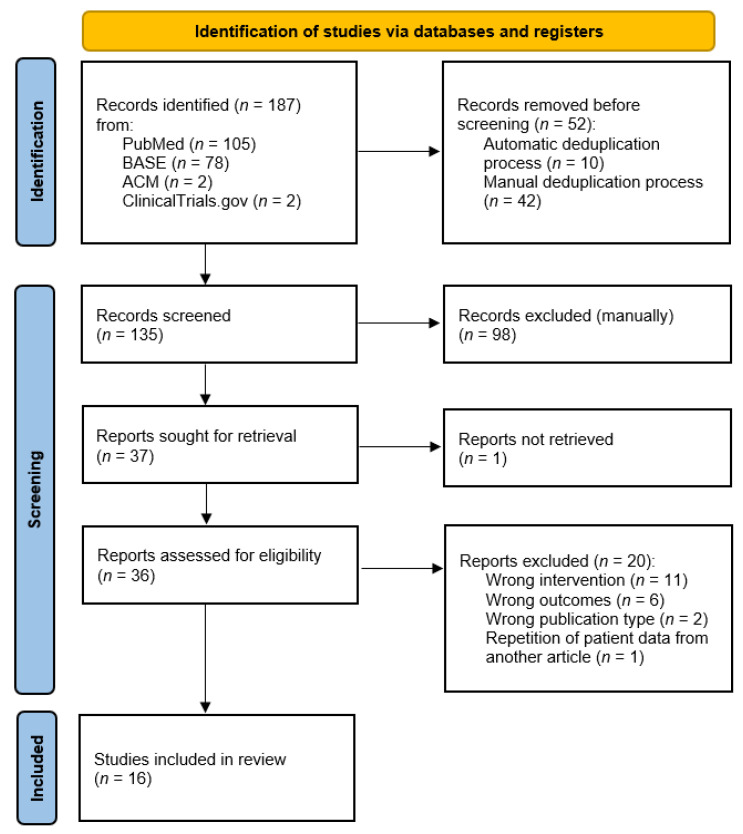
PRISMA flow diagram showing the stages of the studies/study selection.

The individual stages of study selection in accordance with the PRISMA methodology are presented in Figure 1. The details on the ratings of individual records can be found in Supplementary S3. The assessment coefficient of compliance of the assessments at the screening stage was *κ* = 0.82, which is determined as strong, but not very strong, convergence [16]. In order to minimize the risk of eligible studies being omitted from the synthesis, all reports that were evaluated inconsistently at the screening stage were processed further. It was not possible to obtain the full text of one report published by Kopp et al. from 1991 [27]. Ultimately, 16 studies qualified for the synthesis.

### 3.2. Study Characteristics

The reports describing the studies qualified for the systematic review are summarized in Table 3. The 20 individual study groups with a total of 1007 patients treated with intra-articular injections of HA are characterized in detail in Table 4.

### 3.3. Risk of Bias in Studies

The risk of bias was assessed using the RoB 2 tool for randomized controlled trials and is presented in Table 5. None of the other prospective trials qualified for risk assessment using the ROBINS-I tool due to the lack of control groups. Retrospective studies and case series reports are not assessed for the risk of bias, and it was assumed that they had a high risk of bias.

### 3.4. Results of Individual Studies

Full consistency was achieved in the data extracted independently by two investigators. The values of variables extracted from the reports and processed for the purposes of this synthesis (averaging the lateral mobility of the mandible) are presented in Table 6. Table 7 shows the effectiveness of HA treatment in individual domains. In total, 100% was assumed as the initial value of a given variable. Pain values are expected to decrease below 100% and mandibular mobility values to increase above 100% with HA treatment.

### 3.5. Results of Syntheses

#### 3.5.1. Pain

**Figure 2:**
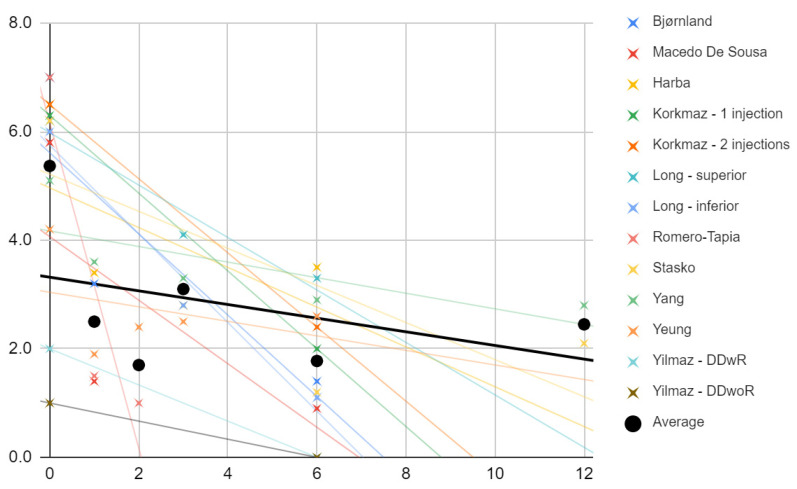
Change in pain intensity (VAS) over time (months). Description in the text.

Figure 2 shows the pain values in each study at individual observation times (crosses), and a trend line was drawn for each study. The slopes of the trend lines clearly indicate that in each of the studies included, there was a decrease in pain intensity as determined by the patients on the VAS scale. The mean values taken from all test values for individual time intervals (dots) are marked in black. An attempt was made to fit a linear regression model (R^2^ = 17%; black line).

#### 3.5.2. Abduction

**Figure 3:**
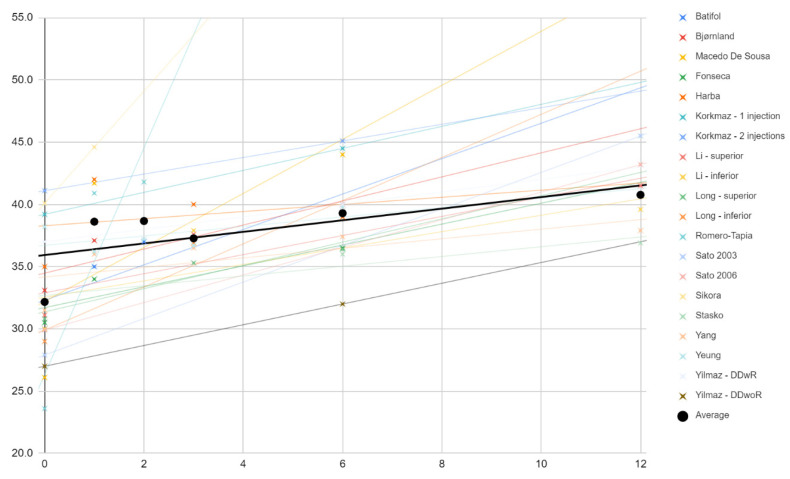
Change in the value of mandibular abduction (mm) over time (months). Description in the text.

Changes in the mouth opening range in the course of individual treatments (crosses) and as the mean (dots) are shown in Figure 3. Trend lines for the studies indicate that each time the extent of mandibular abduction increased as a result of the therapy. The means of these measurements (black) can fit roughly (R^2^ = 48%) to the linear regression model expressed by the formula:

0.5*x* + 36

#### 3.5.3. Protrusive Movement

**Figure 4:**
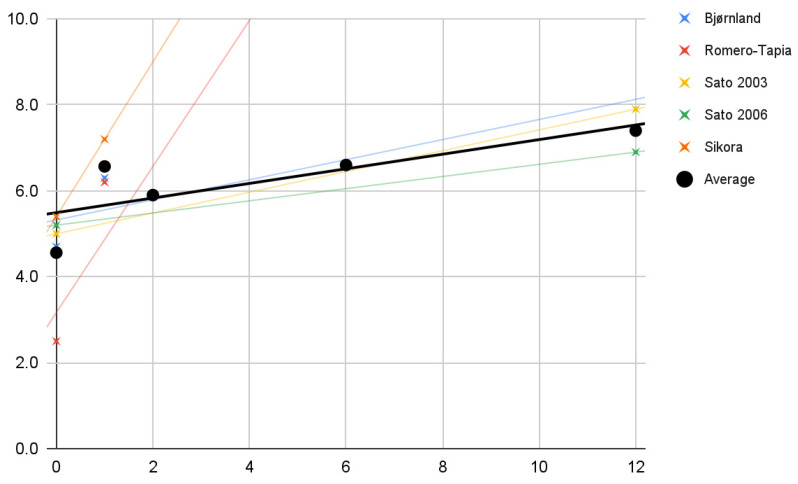
Changes in the range of mandibular protrusive movement (mm) over time (months). Description in the text.

The values of the mandible’s protrusive movement amplitude in the studies of individual authors (crosses) are plotted on the coordinate axis in Figure 4. Again, only the improvement of the parameter was observed. Despite the few studies taking into account the study of mandibular mobility forward, it is possible to notice a fairly good (R^2^ = 62%) fit of the linear regression model (black line) to the mean of the values for individual studies (dots). The trend lines for studies with a longer observation period have a very similar course to this model. This model can be expressed by the formula:

0.2*x* + 5.5

#### 3.5.4. Lateral Movements

**Figure 5:**
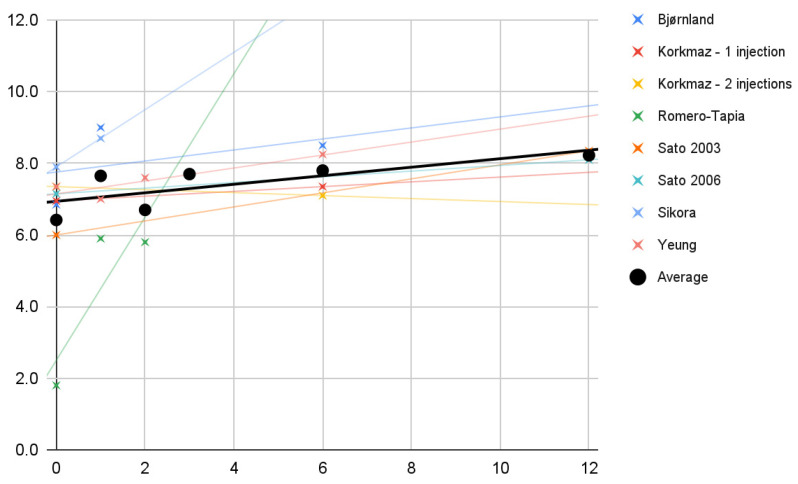
Changes in the average range of mandibular lateral movements (mm) over time (months). Description in the text.

The averaged values of the lateral mandible mobility for individual tests are shown graphically with crosses (Figure 5). Most of their trend lines show a slight improvement with HA treatment. The average values of changes in the mandible’s lateral movements’ range (dots) fit fairly/quite well (R^2^ = 57%) into the linear regression model expressed by the formula:

0.1*x* + 6.9

### 3.6. Correlations

The above-mentioned data characterizing the study groups and the values of the variables concerning pain and mandibular mobility were examined for the presence of correlation. The results are presented in Table 8. The results, which are statistically significant according to the assumptions of the analysis (*p* < 0.05), are bolded.

#### 3.6.1. Pain

The severity of pain 6 months after the beginning of treatment positively correlates with the number of injections per joint (0.63), the total amount of drug administered in milliliters (0.62), and the volume of a drug administered monthly per joint (0.50). These correlations are not strong, but they are enough to raise suspicions whether repeated interventions reduce the analgesic effect, i.e., fewer administrations of HA may be more effective in managing TMJ pain. The intensity of pain after 6 months, starting from the beginning of the therapy, depends, to some extent (0.45), on the initial pain value as well. This means that the stronger the pain before starting the treatment, the greater its severity may remain during the follow-up period.

#### 3.6.2. Abduction

There is a stronger than the previous negative correlation (−0.70) of the increase in the maximum opening of the mouth after 6 months from the beginning of the treatment with the initial values of this variable. Therefore, it can be assumed that the stronger the restriction of vertical mobility of the mandible, the greater the effect in this domain will be achieved by HA therapy. Weak negative correlations are observed between the increase in the maximum mandibular abduction after six months from the beginning of treatment and the following values: (1) ml of the drug per injection (−0.34); (2) number of injections per joint (−0.24); (3) total amount of drug administered per joint expressed in ml (−0.30); (4) the number of ml of drug administered monthly per joint (−0.28). For the four correlations mentioned, the *p* coefficient did not reach the required value. However, the possibility that a greater supply of HA reduces the effectiveness of the therapy should be taken into account.

### 3.7. Possible Causes of Heterogeneity

Good treatment results in the reports of Macedo De Sousa et al. may result from simultaneous splint therapy [4]. Romero-Tapia et al. describe a study to which they qualified only patients with a high degree of mandible mobility limitation, which could have contributed to the impressive treatment effect [35].

## 4. Discussion

### 4.1. HA vs. Stabilization Splint

The efficacy of intra-articular injections of HA versus the use of stabilizing splints was compared by Korkmaz et al. [32]. These authors demonstrated the superiority of HA therapy both in the domain of mandibular mobility and in pain relief [32].

### 4.2. HA vs. Arthrocentesis

Yilmaz et al. compared the effectiveness of HA therapy with the effectiveness of HA administration preceded by arthrocentesis in groups of patients diagnosed with disk displacement with and without reduction. In both diagnoses, the effectiveness of the combination therapy was noticeably higher [41].

### 4.3. HA vs. Blood Products

Macedo De Sousa et al. demonstrated a better immediate effect of HA supply combined with splint therapy than splint therapy alone or intra-articular PRP injections combined with splint therapy [4]. This effect was determined from pain measurements and the extent of mouth opening [4]. After 6 months of follow-up, the PRP-treated group had noticeably better results in the domains of pain relief and increasing the amplitude of mandibular abduction [4]. Harba et al. compared the effectiveness of HA and HA in combination with PRP [31]. A study by these authors showed that combination therapy of HA with PRP brings greater pain relief and gives better results in the domain of mandibular abduction [31].

### 4.4. HA vs. Steroids

Batifol et al. demonstrated higher effectiveness of HA therapy compared to intra-articular injections of corticosteroids in the 6-month follow-up period, both in the domain of mandibular mobility and pain [28]. Similar results were achieved by Bjørnland et al., who demonstrated much higher effectiveness of HA in pain relief compared to corticosteroids [29]. On the other hand, Romero-Tapia et al. did not observe significant differences in the effectiveness of these therapies during the 2-month follow-up period [35].

### 4.5. Limitations of the Evidence

#### 4.5.1. Patient Description

In some studies, patient groups were heterogeneous in terms of diagnosis. In future reviews involving subsequent reports, it is worth considering subgrouping patients with the following diagnoses in particular: (1) disk displacement with reduction; (2) disk displacement without reduction; (3) osteoarthritis, and (4) degenerative joint disease. Such division may help in assessing the effectiveness of HA therapy in individual patient groups.

#### 4.5.2. Intervention Description

The studies differed in the compartment of the joint into which the HA was administered. Assessment of the effectiveness of HA therapy depending on the administration to the upper or lower part of the joint cavity (above or below the articular disc) is a significant problem and should be considered in future systematic reviews based on more reports. In addition, various drugs containing HA in different concentrations have been used, which further complicates the formulation of strong conclusions.

#### 4.5.3. Comparators Description

In this systematic review, baseline values for the variables of mobility and pain were taken as reference values. The efficacy of HA treatment compared to alternative therapies was addressed in the discussion. More high-quality studies comparing HA treatment with placebo with alternative treatments are needed, especially arthrocentesis and intra-articular administration of blood products.

#### 4.5.4. Outcomes Description

The synthesized studies differed with regard to the method of measuring the amplitude of the mandible abduction. The authors of this systematic review are not aware of any studies indicating the most appropriate method of measuring this value. For the purposes of the synthesis, it was assumed that in the case of varying methods of measuring the extent of mandible abduction, the maximum value of the opening that is unsupported manually should be used, and then the value of the maximum pain-free mouth opening. Only in the absence of the above-mentioned data, the value of the manually assisted opening of the mouth was used.

### 4.6. Limitations of the Review Processes

#### 4.6.1. Settings

Only the reports published in English were eligible for systematic review.

#### 4.6.2. Information Sources

Due to the transparency and repeatability of the selection process, it was decided to search only the databases with free access. This could have resulted in the omission of reports indexed only in commercial databases, in particular those kept by publishers.

## 5. Conclusions

The effectiveness of the administration of hyaluronic acid into the temporomandibular joints’ cavities, used as monotherapy, was assessed in 20 study groups with a total of over 1000 patients. The increase in the amplitude of mandibular abduction was expressed as the quotient of the mean values during the observation periods, and the initial value was achieved in all study groups, and in the linear regression model, it was 0.5 mm on average per month. The increase in the mouth opening range is probably inversely proportional to the initial value of this parameter. The mean value of pain in the temporomandibular joints decreased in each of the study groups over the course of the therapy. Multiple administrations of the drug may reduce the analgesic effectiveness of the treatment.

## Figures and Tables

**Table 1:** Criteria for including studies in the systematic review.

Criterion	Inclusion Criteria	Exclusion Criteria
Patient description	Patients diagnosed with pain in the temporomandibular joint according to ICOP Section 3 [1]	Animal patients
Intervention description	Administration of HA into the TMJ cavity	Concomitant other TMJ interventions (e.g., arthroscopy, arthrocentesis) or drug treatment of TMJ other than acute pain relief
Comparator description	Any or none	-
Outcome description	Primary outcomes: mandible abduction ranges Secondary outcomes: horizontal ranges of mandibular mobility and TMJ pain intensity values	No values of mandibular abduction measured before and after injection or series of injections
Settings	Primary studies with a minimum of 10 patients in the HA treatment group	Reports in languages other than English

**Table 2:** Search strategies.

Database	Database Query
ACM	[[All: temporomandibular] OR [All: tmj] OR [All: tmd]] AND [[All: hyaluronic] OR [All: hyaluronan] OR [All: hyaluronate] OR [All: viscosupplement]] AND [[All: injection] OR [All: administration] OR [All: viscosupplementation]] AND [[All: mouth] OR [All: jaw] OR [All: mandible] OR [All: mandibular]] AND [[All: opening] OR [All: abduction] OR [All: mobility] OR [All: protrusion] OR [All: movement]]
BASE	(temporomandibular tmj tmd) AND (hyaluronic hyaluronan hyaluronate viscosupplement) AND (injection administration viscosupplementation) AND (mouth jaw mandible mandibular) AND (opening abduction mobility protrusion movement)
ClinicalTrials.gov	(hyaluronic OR hyaluronan OR hyaluronate OR viscosupplement) AND (injection OR administration OR viscosupplementation) AND (mouth OR jaw OR mandible OR mandibular) AND (opening OR abduction OR mobility OR protrusion OR movement) | Completed Studies | Studies With Results | (temporomandibular OR tmj OR tmd)
PubMed	(temporomandibular OR tmj OR tmd) AND (hyaluronic OR hyaluronan OR hyaluronate OR viscosupplement) AND (injection OR administration OR viscosupplementation) AND (mouth OR jaw OR mandible OR mandibular) AND (opening OR abduction OR mobility OR protrusion OR movement)

**Table 3:** Reports describing studies that meet the eligibility criteria.

First Author	Publication Year	Title	Type of Study
Batifol [28]	2018	The Effect of Intra-Articular Injection of Hyaluronic Acid on the Degenerative Pathology of the Temporo-Mandibular Joint	Retrospective
Bjørnland [29]	2007	Osteoarthritis of the temporomandibular joint: an evaluation of the effects and complications of corticosteroid injection compared with injection with sodium hyaluronate	Randomized controlled trial
Macedo De Sousa [4]	2020	Different Treatments in Patients with Temporomandibular Joint Disorders: A Comparative Randomized Study	Randomized controlled trial
Fonseca [30]	2018	Effectiveness of Sequential Viscosupplementation in Temporomandibular Joint Internal Derangements and Symptomatology: A Case Series	Case series
Harba [31]	2021	Evaluation of the participation of hyaluronic acid with platelet-rich plasma in the treatment of temporomandibular joint disorders	Randomized controlled trial
Korkmaz [32]	2016	Is Hyaluronic Acid Injection Effective for the Treatment of Temporomandibular Joint Disc Displacement With Reduction?	Randomized controlled trial
Li [33]	2015	Osteoarthritic changes after superior and inferior joint space injection of hyaluronic acid for the treatment of temporomandibular joint osteoarthritis with anterior disc displacement without reduction: a cone-beam computed tomographic evaluation	Randomized controlled trial
Long [34]	2009	A randomized controlled trial of superior and inferior temporomandibular joint space injection with hyaluronic acid in treatment of anterior disc displacement without reduction	Randomized controlled trial
Romero-Tapia [35]	2020	Therapeutic Effect of Sodium Hyaluronate and Corticosteroid Injections on Pain and Temporomandibular Joint Dysfunction: A Quasi-experimental Study	Randomized controlled trial
Sato [36]	2003	Analysis of kinesiograph recordings and masticatory efficiency after treatment of non-reducing disk displacement of the temporomandibular joint	Prospective, non-randomized
Sato [37]	2006	Changes in condylar mobility and radiographic alterations after treatment in patients with non-reducing disc displacement of the temporomandibular joint	Prospective, non-randomized
Sikora [3]	2020	Short-Term Effects of Intra-Articular Hyaluronic Acid Administration in Patients with Temporomandibular Joint Disorders	Prospective, non-randomized
Stasko [38]	2020	Hyaluronic acid application vs. arthroscopy in treatment of internal temporomandibular joint disorders	Retrospective
Yang [39]	2018	Oral Glucosamine Hydrochloride Combined With Hyaluronate Sodium Intra-Articular Injection for Temporomandibular Joint Osteoarthritis: A Double-Blind Randomized Controlled Trial	Randomized controlled trial
Yeung [40]	2006	Short-term therapeutic outcome of intra-articular high molecular weight hyaluronic acid injection for non-reducing disc displacement of the temporomandibular joint	Prospective, non-randomized
Yilmaz [41]	2019	Comparison of treatment efficacy between hyaluronic acid and arthrocentesis plus hyaluronic acid in internal derangements of temporomandibular joint	Randomized controlled trial

**Table 4:** Characteristics of the study groups qualified for synthesis. N/A—not applicable; N/S—not specified; DDwR—disk displacement with reduction; DDwoR—disk displacement without reduction.

First Author—Study Group	Trade HA Name	HA per Injection, mL	HA Injections/Joint	Total HA Injected/Joint, mL	Treatment Duration, Weeks	Mean Injection Interval, Weeks	HA Injected Monthly/Joint, mL	Other Interventions	Study Group Size	Diagnosis	Number of Joints Treated	Number of Right Joints Treated	Number of Left Joints Treated	Joints Treated/Patient (Mean)
Batifol	Arthrum	1.0	1	1.0	1	N/A	1.0	None	310	N/S	500	N/S	N/S	1.6
Bjørnland	Synvisc Hylan G-F 20	0.7–1.0	2	2.0	2	2	1.4–2.0	None	20	N/S	20	N/S	N/S	1.0
MacedoDe Sousa	Hyalart	1.0	1	1.0	N/A	N/A	1.0	Bite splint	20	N/S	20	N/S	N/S	1.0
Fonseca	Polireumin/Osteonil Mini	1.0	4	4.0	16	4	1.0	None	10	DDwR	20	10	10	2.0
Harba	Hyalgan	1.0	4	4.0	8	2	2.0	None	12	N/S	N/S	N/S	N/S	N/S
Korkmaz—1 injection	Orthovisc	1.0	1	1.0	N/A	N/A	1.0	None	13	DDwR	20	10	10	1.5
Korkmaz—2 injections	Orthovisc	1.0	2	2.0	4	4	2.0	None	13	DDwR	15	10	5	1.2
Li—superior	SJFBP	1.0	3	3.0	6	2	2.0	None	73	DDwoR	73	43	30	1.0
Li—inferior	SJFBP	1.0	3	3.0	6	2	2.0	None	68	DDwoR	68	37	31	1.0
Long—superior	SJFBP	1.0	3	3.0	6	2	2.0	None	50	DDwoR	60	32	28	1.2
Long—inferior	SJFBP	1.0	3	3.0	6	2	2.0	None	54	DDwoR	66	30	36	1.2
Romero-Tapia	Suprahyal	1.0	1	1.0	N/A	N/A	1.0	None	15	N/S	15	10	5	1.0
Sato 2003	Artz	1.0	5	5.0	5	1	4.0	None	20	DDwoR	20	N/S	N/S	1.0
Sato 2006	Artz	1.0	5	5.0	5	1	4.0	None	55	DDwoR	55	N/S	N/S	1.0
Sikora	Synocrom	0.4	3–5	1.84	3–5	1	1.6	None	40	N/S	61	N/S	N/S	1.5
Stasko	Sinovial Mini	1.0	3	3.0	3	1	3.0	None	99	N/S	99	51	48	1.0
Yang	Sofast	2.0	4	8.0	4	1	8.0	None	72	N/S	87	N/S	N/S	1.2
Yeung	Synvisc Hylan G-F 20	2.0	2	4.0	2	2	4.0	None	27	DDwoR	34	16	18	1.3
Yilmaz—DDwR	Orthovisc	2.0	1	2.0	N/A	N/A	2.0	None	18	DDwR	22	9	13	1.2
Yilmaz—DDwoR	Orthovisc	2.0	1	2.0	N/A	N/A	2.0	None	18	DDwoR	25	12	13	1.4

**Table 5:** The assessment of the risk of bias in individual studies. N/A—not applicable.

First Author	Publication Year	Randomization Process	Deviations of Intended Interventions	Missing Outcome Data	Measurement of Outcomes	Selection of Reported Results	Overall Risk of Bias
Bjørnland	2007	Low	Moderate	Low	Moderate	Low	Moderate
Macedo De Sousa	2020	Moderate	Moderate	Low	Moderate	Low	Moderate
Harba	2021	Moderate	Moderate	Low	Moderate	Low	Moderate
Korkmaz	2016	Moderate	Moderate	Low	Low	Low	Moderate
Li	2015	Moderate	Moderate	Moderate	Low	Low	Moderate
Long	2009	Moderate	Moderate	Low	Moderate	Low	Moderate
Romero-Tapia	2020	High	Moderate	Low	Moderate	Low	Moderate
Yang	2018	Low	Low	Low	Low	Low	Low
Yilmaz	2019	Moderate	Moderate	Low	Low	Low	Moderate

**Table 6:** Collective presentation of the results of studies included in the systematic review.

First Author—Study Group	Pain Values (VAS) in Months						Maximum Mouth Opening (mm) in Months						Protrusive Movement (mm) in Months					Lateral Movements (mm) in Months					
	0	1	2	3	6	12	0	1	2	3	6	12	0	1	2	6	12	0	1	2	3	6	12
Batifol							30.0	35.0	37.0		40.0												
Bjørnland	7.0	3.2			1.4		33.1	37.1			40.0		4.7	6.3		6.6		6.9	9.0			8.5	
Macedo De Sousa	5.8	1.4			0.9		26.1	41.7			44.0												
Fonseca							30.5	34.0			36.5												
Harba	6.5	3.4		2.8	3.5		35.0	42.0		40.0	39.0												
Korkmaz—1 injection	6.3				2.0		39.2				44.5							7.0				7.4	
Korkmaz—2 injections	6.5				2.4		41.1				45.1							7.4				7.1	
Li—superior							31.1			37.6		41.5											
Li—inferior							30.0			37.9		39.6											
Long—superior	6.2			4.1	3.3		30.8			35.3	36.4												
Long—inferior	6.0			2.8	1.1		29.0			36.9	39.4												
Romero-Tapia	7.0	1.5	1.0				23.6	40.9	41.8				2.5	6.2	5.9			1.8	5.9	5.8			
Sato 2003							27.9					45.5	5.0				7.9	6.0					8.4
Sato 2006							29.9					43.2	5.2				6.9	7.2					8.1
Sikora							40.1	44.6					5.4	7.2				7.9	8.7				
Stasko	6.2				1.2	2.1	32.2				36.0	36.9											
Yang	5.1	3.6		3.3	2.9	2.8	31.5	36.0		36.5	37.4	37.9											
Yeung	4.2	1.9	2.4	2.5	2.6		38.2	36.2	37.2	36.7	39.8							7.4	7.0	7.6	7.7	8.3	
Yilmaz—DDwR	2.0				0.0		37.0				40.0												
Yilmaz—DDwoR	1.0				0.0		27.0				32.0												
Average	5.3	2.3	1.7	3.2	1.6	2.5	32.0	38.2	38.7	36.8	39.3	40.8	4.6	6.6	5.9	6.6	7.4	6.4	7.7	6.7	7.7	7.8	8.2
Median	6.1	1.9	1.7	3.1	1.4	2.5	30.8	36.7	37.2	36.8	39.8	40.6	5.0	6.3	5.9	6.6	7.4	7.1	7.9	6.7	7.7	7.8	8.2
Standard deviation	1.9	1.0	1.0	0.7	1.1	0.5	4.9	3.7	2.7	0.9	3.7	3.3	1.2	0.6			0.7	1.9	1.5	1.3		0.7	0.2

**Table 7:** The effectiveness of HA therapies conducted by various researchers.

First Author—Study Group	Pain Values (VAS) in Months						Maximum Mouth Opening (mm) in Months						Protrusive Movement (mm) in Months					Lateral Movements (mm) in Months					
	0	1	2	3	6	12	0	1	2	3	6	12	0	1	2	6	12	0	1	2	3	6	12
Batifol							100%	117%	123%		133%												
Bjørnland	100%	46%			20%		100%	112%			121%		100%	134%		140%		100%	131%			124%	
Macedo De Sousa	100%	24%			16%		100%	160%			169%												
Fonseca							100%	111%			120%												
Harba	100%	52%		43%	54%		100%	120%		114%	111%												
Korkmaz—1 injection	100%				32%		100%				114%							100%				106%	
Korkmaz—2 injections	100%				37%		100%				110%							100%				97%	
Li—superior							100%			121%		133%											
Li—inferior							100%			126%		132%											
Long—superior	100%			66%	53%		100%			115%	118%												
Long—inferior	100%			47%	18%		100%			127%	136%												
Romero-Tapia	100%	21%	14%				100%	173%	177%				100%	248%	236%			100%	328%	322%			
Sato 2003							100%					163%	100%				158%	100%					139%
Sato 2006							100%					144%	100%				133%	100%					113%
Sikora							100%	111%					100%	133%				100%	110%				
Stasko	100%				19%	34%	100%				112%	115%											
Yang	100%	71%		65%	57%	55%	100%	114%		116%	119%	120%											
Yeung	100%	45%	57%	60%	62%		100%	95%	97%	96%	104%							100%	95%	103%	105%	112%	
Yilmaz—DDwR	100%				0%		100%				108%												
Yilmaz—DDwoR	100%				0%		100%				119%												
Average	100%	41%	36%	59%	29%	44%	100%	124%	133%	117%	122%	135%	100%	172%	236%	140%	145%	100%	166%	213%	105%	110%	126%

**Table 8:** Correlation matrix. Statistically significant results are shown in bold. Description in the text.

	HA per Injection, mL	HA Injections/Joint	Total HA Injected/Joint, mL	Treatment Duration, Weeks	Mean Injection Interval, Weeks	HA Injected Monthly/Joint, mL	Study Group Size	Number of Joints Treated	Number of Right Joints Treated	Number of Left Joints Treated	Joints Treated/Patient (Mean)	Initial Pain	Pain after 6 Months	Initial Opening	Opening after 6 Months
HA per injection, mL	x														
HA injections/joint	−0.28	x													
Total HA injected/joint, mL	0.34	**0.75**	x												
Treatment duration, weeks	−0.16	**0.47**	0.21	x											
Mean injection interval, weeks	−0.06	−0.43	−0.35	**0.55**	x										
HA injected monthly/joint, mL	**0.50**	**0.49**	**0.89**	−0.25	**−0.51**	x									
Study group size	−0.10	−0.12	−0.10	−0.41	**−0.54**	−0.01	x								
Number of joints treated	−0.10	−0.15	−0.14	−0.36	**−0.58**	−0.08	**0.99**	x							
Number of right joints treated	−0.40	**0.59**	0.40	−0.36	**−0.85**	0.37	**0.99**	**0.99**	x						
Number of left joints treated	−0.26	**0.57**	**0.46**	−0.36	**−0.91**	**0.46**	**0.96**	**0.98**	**0.94**	x					
Joints treated/patient (mean)	0.03	−0.06	−0.09	**0.53**	**0.53**	−0.26	0.16	0.27	**−0.55**	**−0.45**	x				
Initial pain	**−0.85**	0.34	−0.06	0.29	0.29	−0.16	0.12	0.12	0.32	0.17	**−0.47**	x			
Pain after 6 months	−0.02	**0.63**	**0.62**	0.20	−0.02	**0.50**	0.12	0.29	0.09	−0.02	0.14	**0.45**	x		
Initial opening	0.00	0.00	−0.05	−0.24	0.27	0.02	−0.15	−0.10	−0.24	−0.21	0.27	0.01	0.34	x	
Opening after 6 months	−0.34	−0.24	−0.30	0.02	−0.07	−0.28	0.20	0.19	0.24	0.34	−0.16	0.17	−0.31	**−0.70**	x

## Data Availability

The review protocol is registered in the PROSPERO database under the number CRD42022301151. Data are contained within the article or Supplementary Materials.

## Supplementary Materials

The following supporting information can be downloaded at: https://www.mdpi.com/article/10.3390/jcm11071901/s1, Supplementary S1: PRISMA 2020 Checklist; Supplementary S2: PRISMA 2020 for Abstracts Checklist; Supplementary S3: Stages of qualifying articles for systematic review.
