# Supplementary material for: The Administration of Hyaluronic Acid into the Temporomandibular Joints’ Cavities Increases the Mandible’s Mobility: A Systematic Review and Meta-Analysis
Source: J Clin Med. 2022 Mar 29;11(7):1901. doi: 10.3390/jcm11071901 (PMC8999958; doi:10.3390/jcm11071901)
Supplement: Supplementary file 1 [file jcm-11-01901-s001.zip › Supplementary S3. Stages of qualifying articles for systematic review hyaluronic acid.pdf]

Title	Year	Authors	Screening	Eligibility
Management of Internal Disc Derangement Using Normal Saline and Sodium Hyaluronate: A Comparative Study.	2021	Singh N and Dubey SK and Bhanawat N and Rai G and Kumar A and Vatsa R	"M.C.": "Excluded", "K.C.": "Excluded", Exclusion reason: wrong drug	Not applicable
[Clinical example of complex diagnostic and treatment of patient with temporomandibular joint internal derangements with arthroscopic surgery].	2021	Shipika DV and Ostashko AA and Burenchev DV and Lyan DV and Drobyshev AU	"M.C.": "Excluded", "K.C.": "Excluded", Exclusion reason: foreign language	Not applicable
Double-Needle Arthrocentesis with Viscosupplementation in Patients with Temporomandibular Joint Disc Displacement without Reduction.	2021	Rossini R and Grossmann E and Poluha RL and Setogutti ÊT and Dos Santos MF	"M.C.": "Excluded", "K.C.": "Excluded", Exclusion reason: wrong drug	Not applicable
Evaluation of the participation of hyaluronic acid with platelet-rich plasma in the treatment of temporomandibular joint disorders.	2021	Harba AN and Harfoush M	"M.C.": "Included", "K.C.": "Included"	"M.C.": "Included", "K.C.": "Included"
The efficacy of intra-articular injectable platelet-rich fibrin application in the management of Wilkes stage III temporomandibular joint internal derangement.	2021	Torul D and Cezairli B and Kahveci K	"M.C.": "Excluded", "K.C.": "Excluded", Exclusion reason: wrong drug	Not applicable
Therapeutic Effect of Sodium Hyaluronate and Corticosteroid Injections on Pain and Temporomandibular Joint Dysfunction: A Quasi-experimental Study.	2020	Romero-Tapia P and Pérez-Vargas F and Sedano-Balbin G and Marín J and Mayta-Tovalino F	"M.C.": "Included", "K.C.": "Included"	"M.C.": "Included", "K.C.": "Included"
Does glucosamine, chondroitin sulfate, and methylsulfonylmethane supplementation improve the outcome of temporomandibular joint osteoarthritis management with arthrocentesis plus intraarticular hyaluronic acid injection. A randomized clinical trial.	2021	Cömert Kılıç S	"M.C.": "Excluded", "K.C.": "Excluded", Exclusion reason: wrong drug	Not applicable
Temporomandibular Joint Arthrocentesis and Microfragmented Adipose Tissue Injection for the Treatment of Internal Derangement and Osteoarthritis: A Randomized Clinical Trial.	2021	Sembronio S and Tel A and Tremolada C and Lazzarotto A and Isola M and Robiony M	"M.C.": "Excluded", "K.C.": "Excluded", Exclusion reason: wrong drug	Not applicable
Is there a difference in treatment effect of different intra-articular drugs for temporomandibular joint osteoarthritis? A systematic review of randomized controlled trials.	2021	Liapaki A and Thamm JR and Ha S and Monteiro JLGC and McCain JP and Troulis MJ and Guastaldi FPS	"M.C.": "Excluded", "K.C.": "Excluded", Exclusion reason: wrong publication type	Not applicable
Arthrocentesis and Sodium Hyaluronate Infiltration in Temporomandibular Disorders Treatment. Clinical and MRI Evaluation.	2020	Santagata M and De Luca R and Lo Giudice G and Troiano A and Lo Giudice G and Corvo G and Tartaro G	"M.C.": "Excluded", "K.C.": "Excluded", Exclusion reason: wrong drug	Not applicable
Treatment of Temporomandibular Joint Arthritis with Triamcinolone Acetonide and Hyaluronic Acid Injection: An Observational Study.	2020	Singh J and Bhardwaj B	"M.C.": "Excluded", "K.C.": "Excluded", Exclusion reason: wrong drug	Not applicable
Is arthrocentesis plus hyaluronic acid superior to arthrocentesis alone in the treatment of disc displacement without reduction in patients with bruxism?	2020	Hosgor H	"M.C.": "Excluded", "K.C.": "Excluded", Exclusion reason: wrong drug	Not applicable
Synovial fluid levels of VEGF and FGF-2 before and after intra-articular injection of hyaluronic acid in patients with temporomandibular disorders: a short-term study.	2021	Wang XW and Fang W and Li YJ and Long X and Cai HX	"M.C.": "Included", "K.C.": "Included"	"M.C.": "Excluded", "K.C.": "Excluded", Exclusion reason: wrong intervention
TMJ arthroscopy with hyaluronic acid: A 12-month randomized clinical trial.	2021	Castaño-Joaqui OG and Cano-Sánchez J and Campo-Trapero J and Muñoz-Guerra MF	"M.C.": "Excluded", "K.C.": "Included"	"M.C.": "Excluded", "K.C.": "Excluded", Exclusion reason: wrong intervention
Short-Term Effects of Intra-Articular Hyaluronic Acid Administration in Patients with Temporomandibular Joint Disorders.	2020	Sikora M and Czerwińska-Niezabitowska B and Chęciński MA and Sielski M and Chlubek D	"M.C.": "Included", "K.C.": "Included"	"M.C.": "Included", "K.C.": "Included"
Platelet Concentrate Treatments for Temporomandibular Disorders: A Systematic Review and Meta-analysis.	2021	Al-Hamed FS and Hijazi A and Gao Q and Badran Z and Tamimi F	"M.C.": "Excluded", "K.C.": "Excluded", Exclusion reason: wrong publication type	Not applicable
Comparison of the Efficiacy of Intra-Articular Injection of Liquid Platelet-Rich Fibrin and Hyaluronic Acid After in Conjunction With Arthrocentesis for the Treatment of Internal Temporomandibular Joint Derangements.	2020	Yuce E and Komerik N	"M.C.": "Excluded", "K.C.": "Excluded", Exclusion reason: wrong drug	Not applicable

Hyaluronic acid application vs arthroscopy in treatment of internal temporomandibular joint disorders.	2020	Stasko J and Statelova D and Janickova M and Mikuskova K and Bacinsky M and Sokol J and Frlickova Z and Hvizdos D and Malachovsky I	"M.C.": "Included", "K.C.": "Included"	"M.C.": "Included", "K.C.": "Included"
Different Treatments in Patients with Temporomandibular Joint Disorders: A Comparative Randomized Study.	2020	Macedo De Sousa BM and López-Valverde N and López-Valverde A and Caramelo F and Fraile JF and Payo JH and Rodrigues MJ	"M.C.": "Included", "K.C.": "Included"	"M.C.": "Included", "K.C.": "Included"
The hierarchy of different treatments for arthrogenous temporomandibular disorders: A network meta-analysis of randomized clinical trials.	2020	Al-Moraissi EA and Wolford LM and Ellis E 3rd and Neff A	"M.C.": "Excluded", "K.C.": "Excluded", Exclusion reason: wrong publication type	Not applicable
Comparison of efficacy of sodium hyaluronate and normal saline arthrocentesis in the management of internal derangement of temporomandibular joints - A prospective study.	2019	Rao JKD and Sharma A and Kashyap R and Walecha K and Siwach V and Arya V	"M.C.": "Excluded", "K.C.": "Excluded", Exclusion reason: wrong drug	Not applicable
Multiple Treatment Meta-Analysis of Intra-Articular Injection for Temporomandibular Osteoarthritis.	2020	Liu Y and Wu JS and Tang YL and Tang YJ and Fei W and Liang XH	"M.C.": "Excluded", "K.C.": "Excluded", Exclusion reason: wrong publication type	Not applicable
Bone marrow nucleated cell concentrate autograft in temporomandibular joint degenerative disorders: 1-year results of a randomized clinical trial.	2019	De Riu G and Vaira LA and Carta E and Meloni SM and Sembronio S and Robiony M	"M.C.": "Excluded", "K.C.": "Excluded", Exclusion reason: wrong drug	Not applicable
Comparison of treatment efficacy between hyaluronic acid and arthrocentesis plus hyaluronic acid in internal derangements of temporomandibular joint.	2019	Yilmaz O and Korkmaz YT and Tuzuner T	"M.C.": "Included", "K.C.": "Included"	"M.C.": "Included", "K.C.": "Included"
Intra-articular injection of a mixture of hyaluronic acid and corticosteroid versus arthrocentesis in TMJ internal derangement.	2020	Marzook HAM and Abdel Razek AA and Yousef EA and Attia AAMM	"M.C.": "Excluded", "K.C.": "Excluded", Exclusion reason: wrong drug	Not applicable
Evaluation of success criteria for temporomandibular joint arthrocentesis.	2019	Yilmaz O and Candirli C and Balaban E and Demirkol M	"M.C.": "Excluded", "K.C.": "Excluded", Exclusion reason: wrong drug	Not applicable
Long-term effectiveness of arthrocentesis with and without hyaluronic acid injection for treatment of temporomandibular joint osteoarthritis.	2019	Bergstrand S and Ingstad HK and Møystad A and Bjørnland T	"M.C.": "Excluded", "K.C.": "Excluded", Exclusion reason: wrong drug	Not applicable
Efficacy of Sodium Hyaluronate for Temporomandibular Joint Disorder by Single-Puncture Arthrocentesis.	2019	Sequeira J and Rao BHS and Kedia PR	"M.C.": "Excluded", "K.C.": "Excluded", Exclusion reason: wrong drug	Not applicable
Use of platelet-rich plasma, platelet-rich growth factor with arthrocentesis or arthroscopy to treat temporomandibular joint osteoarthritis: Systematic review with meta-analyses.	2018	Haigler MC and Abdulrehman E and Siddappa S and Kishore R and Padilla M and Enciso R	"M.C.": "Excluded", "K.C.": "Excluded", Exclusion reason: wrong publication type	Not applicable
Evaluation of Effect of Glucosamine-Chondroitin Sulfate, Tramadol, and Sodium Hyaluronic Acid on Expression of Cytokine Levels in Internal Derangement of Temporomandibular Joint.	2018	Ganti S and Shriram P and Ansari AS and Kapadia JM and Azad A and Dubey A	"M.C.": "Included", "K.C.": "Included"	"M.C.": "Excluded", "K.C.": "Excluded", Exclusion reason: wrong intervention
Platelet-Rich Plasma in Treatment of Temporomandibular Joint Dysfunctions: Narrative Review.	2019	Zotti F and Albanese M and Rodella LF and Nocini PF	"M.C.": "Excluded", "K.C.": "Excluded", Exclusion reason: wrong publication type	Not applicable
Effectiveness of platelet-rich plasma injection in patients with temporomandibular joint osteoarthritis: a systematic review and meta-analysis of randomized controlled trials.	2019	Chung PY and Lin MT and Chang HP	"M.C.": "Excluded", "K.C.": "Excluded", Exclusion reason: wrong publication type	Not applicable
[Intra-articular steroid and hyaluronic acid treatment of internal derangement of the temporomandibular joint].	2018	Vingender S and Restár L and Csomó KB and Schmidt P and Hermann P and Vaszilkó M	"M.C.": "Excluded", "K.C.": "Excluded", Exclusion reason: foreign language	Not applicable

Effectiveness of Sequential Viscosupplementation in Temporomandibular Joint Internal Derangements and Symptomatology: A Case Series.	2018	Fonseca RMDFB and Januzzi E and Ferreira LA and Grossmann E and Carvalho ACP and de Oliveira PG and Vieira ÉLM and Teixeira AL and Almeida-Leite CM	"M.C.": "Included", "K.C.": "Included"	"M.C.": "Included", "K.C.": "Included"
Prediction Models for Oral Health-Related Quality of Life in Patients with Temporomandibular Joint Osteoarthritis 1 and 6 Months After Arthrocentesis with Hyaluronic Acid Injections.	2019	Su N and Wang H and van Wijk AJ and Visscher CM and Lobbezoo F and Shi Z and van der Heijden GJMG	"M.C.": "Excluded", "K.C.": "Excluded", Exclusion reason: wrong drug	Not applicable
Clinical Outcome of Sodium Hyaluronate Injection into the Superior and Inferior Joint Space for Osteoarthritis of the Temporomandibular Joint Evaluated by Cone-Beam Computed Tomography: A Retrospective Study of 51 Patients and 56 Joints.	2018	Sun H and Su Y and Song N and Li C and Shi Z and Li L	"M.C.": "Included", "K.C.": "Included"	"M.C.": "Excluded", "K.C.": "Excluded", Exclusion reason: wrong intervention
Effect of intra-articular Botulinum toxin injections on temporo-mandibular joint pain.	2018	Batifol D and Huart A and Finiels PJ and Nagot N and Jammet P	"M.C.": "Excluded", "K.C.": "Excluded", Exclusion reason: wrong drug	Not applicable
Oral Glucosamine Hydrochloride Combined With Hyaluronate Sodium Intra-Articular Injection for Temporomandibular Joint Osteoarthritis: A Double-Blind Randomized Controlled Trial.	2018	Yang W and Liu W and Miao C and Sun H and Li L and Li C	"M.C.": "Included", "K.C.": "Included"	"M.C.": "Included", "K.C.": "Included"
A comparison of the effects of Methylprednisolone Acetate, Sodium Hyaluronate and Tenoxicam in the treatment of non-reducing disc displacement of the temporomandibular joint.	2018	Yapici-Yavuz G and Şimşek-Kaya G and Oğul H	"M.C.": "Excluded", "K.C.": "Excluded", Exclusion reason: wrong drug	Not applicable
Is There a Difference in Intra-Articular Injections of Corticosteroids, Hyaluronate, or Placebo for Temporomandibular Osteoarthritis?	2018	Liu Y and Wu J and Fei W and Cen X and Xiong Y and Wang S and Tang Y and Liang X	"M.C.": "Excluded", "K.C.": "Excluded", Exclusion reason: wrong publication type	Not applicable
Platelet-rich plasma for the therapeutic management of temporomandibular joint disorders: a systematic review.	2018	Bousnaki M and Bakopoulou A and Koidis P	"M.C.": "Excluded", "K.C.": "Excluded", Exclusion reason: wrong publication type	Not applicable
Efficacy of Temporomandibular Joint Arthrocentesis with Sodium Hyaluronate in the Management of Temporomandibular Joint Disorders: A Prospective Randomized Control Trial.	2017	Gorrela H and Prameela J and Srinivas G and Reddy BVB and Sudhir M and Arakeri G	"M.C.": "Excluded", "K.C.": "Excluded", Exclusion reason: wrong drug	Not applicable
Glucosamine oral administration as an adjunct to hyaluronic acid injection in treating temporomandibular joint osteoarthritis.	2018	Cen X and Liu Y and Wang S and Yang X and Shi Z and Liang X	"M.C.": "Included", "K.C.": "Included"	"M.C.": "Excluded", "K.C.": "Excluded", Exclusion reason: repetition of patient data from another article (Yang et al. 2018)
Efficacy of arthrocentesis versus arthrocentesis with sodium hyaluronic acid in temporomandibular joint osteoarthritis: A comparison.	2017	Gurung T and Singh RK and Mohammad S and Pal US and Mahdi AA and Kumar M	"M.C.": "Excluded", "K.C.": "Excluded", Exclusion reason: wrong drug	Not applicable
Age, gender and parafunctional habits as prognostic factors for temporomandibular joint arthrocentesis.	2018	Attia HS and Mosleh MI and Jan AM and Shawky MM and Jadu FM	"M.C.": "Excluded", "K.C.": "Excluded", Exclusion reason: wrong drug	Not applicable
Comparison of intra-articular injection of plasma rich in growth factors versus hyaluronic acid following arthroscopy in the treatment of temporomandibular dysfunction: A randomised prospective study.	2017	Fernández-Ferro M and Fernández-Sanromán J and Blanco-Carrión A and Costas-López A and López-Betancourt A and Arenaz-Bua J and Stavaru Marinescu B	"M.C.": "Included", "K.C.": "Included"	"M.C.": "Excluded", "K.C.": "Excluded", Exclusion reason: wrong intervention
The impact of arthrocentesis with and without hyaluronic acid injection in the prognosis and synovial fluid myeloperoxidase levels of patients with painful symptomatic internal derangement of temporomandibular joint: a randomised controlled clinical trial.	2017	Ozdamar SM and Alev B and Yarat A	"M.C.": "Excluded", "K.C.": "Excluded", Exclusion reason: wrong drug	Not applicable
Sodium hyaluronate: an effective adjunct in temporomandibular joint arthrocentesis.	2016	Patel P and Idrees F and Newaskar V and Agrawal D	"M.C.": "Excluded", "K.C.": "Excluded", Exclusion reason: wrong drug	Not applicable

[Different types of injection in temporomandibular disorders (TMD) treatment].	2016	Batifol D	"M.C.": "Excluded", "K.C.": "Excluded", Exclusion reason: foreign language	Not applicable
[Arthrocentesis of the temporomandibular joint and intra-articular injections : An update].	2016	Marty P and Louvrier A and Weber E and Dubreuil PA and Chatelain B and Meyer C	"M.C.": "Excluded", "K.C.": "Excluded", Exclusion reason: foreign language	Not applicable
Removal of a Solitary Synovial Chondromatosis of the Temporomandibular Joint Using Arthroscopy.	2016	Pastore GP and Goulart DR and Pastore PR and Prati AJ	"M.C.": "Excluded", "K.C.": "Excluded", Exclusion reason: wrong publication type	Not applicable
Is Hyaluronic Acid Injection Effective for the Treatment of Temporomandibular Joint Disc Displacement With Reduction?	2016	Korkmaz YT and Altıntas NY and Korkmaz FM and Candırlı C and Coskun U and Durmuslar MC	"M.C.": "Included", "K.C.": "Included"	"M.C.": "Included", "K.C.": "Included"
Long-Term Outcome of Arthrocentesis Plus Hyaluronic Acid Injection in Patients With Wilkes Stage II and III Temporomandibular Joint Internal Derangement.	2015	Ungor C and Atasoy KT and Taskesen F and Pirpir C and Yilmaz O	"M.C.": "Excluded", "K.C.": "Excluded", Exclusion reason: wrong drug	Not applicable
[Effect of articular cavity injection for patients with temporomandibular joint osteoarthritis at different ages].	2015	Li T and Li G	"M.C.": "Excluded", "K.C.": "Excluded", Exclusion reason: foreign language	Not applicable
Joint cavity injection combined with manual reduction and stabilization splint treatment of anterior disc displacement.	2015	Liu J and Mu H and Wang Z and Lan J and Zhang S and Long X and Zhang D	"M.C.": "Included", "K.C.": "Included"	"M.C.": "Excluded", "K.C.": "Excluded", Exclusion reason: wrong intervention
Comparison Between Betamethasone and Sodium Hyaluronate Combination with Betamethasone Alone After Arthrocentesis in the Treatment of Internal Derangement of TMJ-Using Single Puncture Technique: A Preliminary Study.	2015	Giraddi GB and Siddaraju A and Kumar A and Jain T	"M.C.": "Excluded", "K.C.": "Excluded", Exclusion reason: wrong drug	Not applicable
Platelet-Rich Plasma Injection as an Effective Treatment for Temporomandibular Joint Osteoarthritis.	2015	Hegab AF and Ali HE and Elmasry M and Khallaf MG	"M.C.": "Included", "K.C.": "Included"	"M.C.": "Excluded", "K.C.": "Excluded", Exclusion reason: wrong intervention
Osteoarthritic changes after superior and inferior joint space injection of hyaluronic acid for the treatment of temporomandibular joint osteoarthritis with anterior disc displacement without reduction: a cone-beam computed tomographic evaluation.	2015	Li C and Long X and Deng M and Li J and Cai H and Meng Q	"M.C.": "Included", "K.C.": "Included"	"M.C.": "Included", "K.C.": "Included"
Comparative treatment outcomes of menopausal and nonmenopausal women after arthrocentesis.	2014	Ungor C and Cezairli B and Taskesen F and Dayisoylu EH and Cizmeci Senel F	"M.C.": "Excluded", "K.C.": "Excluded", Exclusion reason: wrong drug	Not applicable
[Treatment of temporomandibular joint disc perforation with injection of hyaluronic acid or disc repair].	2014	Lu J and Long X and Deng M and Cheng Y and Li B	"M.C.": "Excluded", "K.C.": "Excluded", Exclusion reason: foreign language	Not applicable
Effectiveness of treatment with viscosupplementation in temporomandibular joints with or without effusion.	2014	Guarda-Nardini L and Rossi A and Ramonda R and Punzi L and Ferronato G and Manfredini D	"M.C.": "Included", "K.C.": "Included"	"M.C.": "Excluded", "K.C.": "Excluded", Exclusion reason: wrong intervention
A comparative study on the impact of intra-articular injections of hyaluronic acid, tenoxicam and betametazon on the relief of temporomandibular joint disorder complaints.	2014	Gencer ZK and Özkiriş M and Okur A and Korkmaz M and Saydam L	"M.C.": "Included", "K.C.": "Included"	"M.C.": "Excluded", "K.C.": "Excluded", Exclusion reason: wrong outcome
Arthrocentesis and temporomandibular joint disorders: clinical and radiological results of a prospective study.	2013	De Riu G and Stimolo M and Meloni SM and Soma D and Pisano M and Sembronio S and Tullio A	"M.C.": "Excluded", "K.C.": "Excluded", Exclusion reason: wrong drug	Not applicable
Internal derangement of temporomandibular joint: an evaluation of effect of corticosteroid injection compared with injection of sodium hyaluronate after arthrocentesis.	2012	Giraddi GB and Siddaraju A and Kumar B and Singh C	"M.C.": "Excluded", "K.C.": "Excluded", Exclusion reason: wrong drug	Not applicable
The next step in the treatment of persistent temporomandibular joint pain following arthrocentesis: a retrospective study of 18 cases.	2014	Emes Y and Arpınar IŞ and Oncü B and Aybar B and Aktaş I and Al Badri N and Atalay B and Işsever H and Yalçın S	"M.C.": "Excluded", "K.C.": "Excluded", Exclusion reason: wrong drug	Not applicable

Epidemiology, diagnosis, and treatment of temporomandibular disorders.	2013	Liu F and Steinkeler A	"M.C.": "Excluded", "K.C.": "Excluded", Exclusion reason: wrong publication type	Not applicable
Two commentaries on interventions for the management of temporomandibular joint osteoarthritis.	2013	Patel DN and Manfredini D	"M.C.": "Excluded", "K.C.": "Excluded", Exclusion reason: wrong publication type	Not applicable
An assessment of the usefulness of jaw kinesiography in monitoring temporomandibular disorders: correlation of treatment-related kinesiographic and pain changes in patients receiving temporomandibular joint injections.	2013	Manfredini D and Favero L and Michieli M and Salmaso L and Cocilovo F and Guarda-Nardini L	"M.C.": "Excluded", "K.C.": "Excluded", Exclusion reason: wrong drug	Not applicable
Comparison of 2 hyaluronic acid drugs for the treatment of temporomandibular joint osteoarthritis.	2012	Guarda-Nardini L and Cadorin C and Frizziero A and Ferronato G and Manfredini D	"M.C.": "Excluded", "K.C.": "Excluded", Exclusion reason: wrong drug	Not applicable
Treatment effectiveness of arthrocentesis plus hyaluronic acid injections in different age groups of patients with temporomandibular joint osteoarthritis.	2012	Guarda-Nardini L and Olivo M and Ferronato G and Salmaso L and Bonnini S and Manfredini D	"M.C.": "Excluded", "K.C.": "Excluded", Exclusion reason: wrong drug	Not applicable
Interventions for the management of temporomandibular joint osteoarthritis.	2012	Macedo De Souza RF and Lovato da Silva CH and Nasser M and Fedorowicz Z and Al-Muharraqi MA	"M.C.": "Excluded", "K.C.": "Excluded", Exclusion reason: wrong publication type	Not applicable
[Glucosamine hydrochloride combined with hyaluronate for temporomandibular joint osteoarthritis: a primary report of randomized controlled trial].	2011	Li C and Jia Y and Zhang Q and Shi Z and Chen H	"M.C.": "Excluded", "K.C.": "Excluded", Exclusion reason: foreign language	Not applicable
Repeated sodium hyaluronate injections following multiple arthrocenteses in the treatment of early stage reducing disc displacement of the temporomandibular joint: a preliminary report.	2012	Tuncel U	"M.C.": "Excluded", "K.C.": "Excluded", Exclusion reason: wrong drug	Not applicable
Two-needle vs. single-needle technique for TMJ arthrocentesis plus hyaluronic acid injections: a comparative trial over a six-month follow up.	2012	Guarda-Nardini L and Ferronato G and Manfredini D	"M.C.": "Excluded", "K.C.": "Excluded", Exclusion reason: wrong drug	Not applicable
[Hyaluronate sodium treatment for internal derangement of temporomandibular joint: a systematic review based on randomized controlled trials].	2011	Li C and Zhang Y and Jia Y and Lü J and Li L and Shi ZD	"M.C.": "Excluded", "K.C.": "Excluded", Exclusion reason: foreign language	Not applicable
A longitudinal study on the osteoarthritic change of the temporomandibular joint based on 1-year follow-up computed tomography.	2012	Lee JY and Kim DJ and Lee SG and Chung JW	"M.C.": "Excluded", "K.C.": "Excluded", Exclusion reason: wrong drug	Not applicable
Conservative treatment of severe limited mouth opening after transtemporal craniotomy.	2011	Qing-Gong M and Si C and Xing L	"M.C.": "Excluded", "K.C.": "Excluded", Exclusion reason: wrong population	Not applicable
Prognostic indicators of the outcome of arthrocentesis with and without sodium hyaluronate injection for the treatment of disc displacement without reduction: a magnetic resonance imaging study.	2010	Aktas I and Yalcin S and Sencer S	"M.C.": "Excluded", "K.C.": "Excluded", Exclusion reason: wrong drug	Not applicable
Single-needle temporomandibular joint arthrocentesis with hyaluronic acid injections. Preliminary data after a five-injection protocol.	2009	Manfredini D and Guarda-Nardini L and Ferronato G	"M.C.": "Excluded", "K.C.": "Excluded", Exclusion reason: wrong drug	Not applicable
Short-term effects of arthrocentesis plus viscosupplementation in the management of signs and symptoms of painful TMJ disc displacement with reduction. A pilot study.	2010	Guarda-Nardini L and Manfredini D and Ferronato G	"M.C.": "Excluded", "K.C.": "Excluded", Exclusion reason: wrong drug	Not applicable
A randomized controlled trial of superior and inferior temporomandibular joint space injection with hyaluronic acid in treatment of anterior disc displacement without reduction.	2009	Long X and Chen G and Cheng AH and Cheng Y and Deng M and Cai H and Meng Q	"M.C.": "Included", "K.C.": "Included"	"M.C.": "Included", "K.C.": "Included"

Efficacy and safety of sodium hyaluronate in the treatment of Wilkes stage II disease.	2008	Oliveras-Moreno JM and Hernandez-Pacheco E and Oliveras-Quintana T and Infante-Cossio P and Gutierrez-Perez JL	"M.C.": "Included", "K.C.": "Included"	"M.C.": "Excluded", "K.C.": "Excluded", Exclusion reason: wrong outcome
Total temporomandibular joint replacement: a clinical case with a proposal for post-surgical rehabilitation.	2008	Guarda-Nardini L and Manfredini D and Ferronato G	"M.C.": "Excluded", "K.C.": "Excluded", Exclusion reason: wrong publication type	Not applicable
Arthrocentesis of the temporomandibular joint: a proposal for a single-needle technique.	2008	Guarda-Nardini L and Manfredini D and Ferronato G	"M.C.": "Excluded", "K.C.": "Excluded", Exclusion reason: wrong drug	Not applicable
Osteoarthritis of the temporomandibular joint: an evaluation of the effects and complications of corticosteroid injection compared with injection with sodium hyaluronate.	2007	Bjørnland T and Gjaerum AA and Møystad A	"M.C.": "Included", "K.C.": "Included"	"M.C.": "Included", "K.C.": "Included"
A one-year case series of arthrocentesis with hyaluronic acid injections for temporomandibular joint osteoarthritis.	2007	Guarda-Nardini L and Stifano M and Brombin C and Salmaso L and Manfredini D	"M.C.": "Excluded", "K.C.": "Excluded", Exclusion reason: wrong drug	Not applicable
Short-term therapeutic outcome of intra-articular high molecular weight hyaluronic acid injection for nonreducing disc displacement of the temporomandibular joint.	2006	Yeung RW and Chow RL and Samman N and Chiu K	"M.C.": "Included", "K.C.": "Included"	"M.C.": "Included", "K.C.": "Included"
Changes in condylar mobility and radiographic alterations after treatment in patients with non-reducing disc displacement of the temporomandibular joint.	2006	Sato S and Kawamura H	"M.C.": "Excluded", "K.C.": "Included"	"M.C.": "Included", "K.C.": "Included"
Conservative treatment of temporomandibular joint osteoarthrosis: intra-articular injection of sodium hyaluronate.	2005	Guarda-Nardini L and Masiero S and Marioni G	"M.C.": "Included", "K.C.": "Included"	"M.C.": "Excluded", "K.C.": "Excluded", Exclusion reason: wrong intervention
[Influence of intra-articular injections of sodium hyaluronate on clinical features and synovial fluid nitric oxide levels of temporomandibular osteoarthritis].	2004	Guarda Nardini L and Oliviero F and Ramonda R and Ferronato G	"M.C.": "Excluded", "K.C.": "Excluded", Exclusion reason: foreign language	Not applicable
Pre- and posttreatment analysis of clinical symptoms of patients with temporomandibular disorders.	2004	Babadağ M and Sahin M and Görgün S	"M.C.": "Excluded", "K.C.": "Included"	"M.C.": "Excluded", "K.C.": "Excluded", Exclusion reason: wrong intervention
[An outcome analysis of two methods of intra-capsular injection of sodium hyaluronate for temporomandibular disorders].	2004	Li XD and Shi ZD and Tian WD	"M.C.": "Excluded", "K.C.": "Excluded", Exclusion reason: foreign language	Not applicable
[Intraarticular injection-dilatation and later lavage plus viscosupplementation of TMJ for the treatment of anterior disc displacement without reduction].	2004	Zhong WQ and Zhou G	"M.C.": "Excluded", "K.C.": "Excluded", Exclusion reason: foreign language	Not applicable
Analysis of kinesiograph recordings and masticatory efficiency after treatment of non-reducing disk displacement of the temporomandibular joint.	2003	Sato S and Nasu F and Motegi K	"M.C.": "Excluded", "K.C.": "Included"	"M.C.": "Included", "K.C.": "Included"
Treatment of patients with arthrosis of the temporomandibular joint by infiltration of sodium hyaluronate: a preliminary study.	2002	Guarda-Nardini L and Tito R and Staffieri A and Beltrame A	"M.C.": "Excluded", "K.C.": "Excluded", Exclusion reason: wrong drug	Not applicable
The efficacy of intra-articular sodium hyaluronate in patients with reducing displaced disc of the temporomandibular joint.	2002	Hepguler S and Akkoc YS and Pehlivan M and Ozturk C and Celebi G and Saracoglu A and Ozpinar B	"M.C.": "Included", "K.C.": "Included"	"M.C.": "Excluded", "K.C.": "Excluded", Exclusion reason: wrong outcome
Pumping injection of sodium hyaluronate for patients with non-reducing disc displacement of the temporomandibular joint: two year follow-up.	2001	Sato S and Oguri S and Yamaguchi K and Kawamura H and Motegi K	"M.C.": "Excluded", "K.C.": "Included"	"M.C.": "Excluded", "K.C.": "Excluded", Exclusion reason: wrong outcome
Effect of pumping with injection of sodium hyaluronate and the other factors related to outcome in patients with non-reducing disk displacement of the temporomandibular joint.	2001	Sato S and Goto S and Kasahara T and Kawamura H and Motegi K	"M.C.": "Excluded", "K.C.": "Included"	"M.C.": "Excluded", "K.C.": "Excluded", Exclusion reason: wrong outcome
Efficacy of temporomandibular joint arthrocentesis with and without injection of sodium hyaluronate in treatment of internal derangements.	2001	Alpaslan GH and Alpaslan C	"M.C.": "Excluded", "K.C.": "Excluded", Exclusion reason: wrong drug	Not applicable

Disc position and morphology in patients with nonreducing disc displacement treated by injection of sodium hyaluronate.	1999	Sato S and Sakamoto M and Kawamura H and Motegi K	"M.C.": "Included", "K.C.": "Included"	"M.C.": "Excluded", "K.C.": "Excluded", Exclusion reason: wrong outcome
Arthroscopic management of temporomandibular closed lock.	1998	Miyamoto H and Sakashita H and Miyata M and Goss AN and Okabe K and Miyaji Y and Sakuma K	"M.C.": "Excluded", "K.C.": "Excluded", Exclusion reason: wrong drug	Not applicable
Intra-articular injection of hyaluronic acid reduces total amounts of leukotriene C4, 6-keto-prostaglandin F1alpha, prostaglandin F2alpha and interleukin-1beta in synovial fluid of patients with internal derangement in disorders of the temporomandibular joint.	1998	Hirota W	"M.C.": "Excluded", "K.C.": "Excluded", Exclusion reason: wrong drug	Not applicable
Pressurized infusion of sodium hyaluronate for closed lock of the temporomandibular joint. Part I: A case study.	1993	Fader KW and Grummons DC and Maijer R and Christensen LV	"M.C.": "Excluded", "K.C.": "Excluded", Exclusion reason: wrong publication type	Not applicable
Short-term effects of intra-articular sodium hyaluronate, glucocorticoid, and saline injections on rheumatoid arthritis of the temporomandibular joint.	1991	Kopp S and Akerman S and Nilner M	"M.C.": "Included", "K.C.": "Included"	Full text not available
Is Hyaluronic Acid or Corticosteroid Superior to Lactated Ringer Solution in the Short-Term Reduction of Temporomandibular Joint Pain After Arthrocentesis? Part 1.	2017	Bouloux GF and Chou J and Krishnan D and Aghaloo T and Kahenasa N and Smith JA and Giannakopoulos H	"M.C.": "Excluded", "K.C.": "Excluded", Exclusion reason: wrong drug	Not applicable
Is Hyaluronic Acid or Corticosteroid Superior to Lactated Ringer Solution in the Short Term for Improving Function and Quality of Life After Arthrocentesis? Part 2.	2017	Bouloux GF and Chou J and Krishnan D and Aghaloo T and Kahenasa N and Smith JA and Giannakopoulos H	"M.C.": "Excluded", "K.C.": "Excluded", Exclusion reason: wrong drug	Not applicable
Successful temporomandibular viscosupplementation protocol in a 47 year-old woman with a 10- year history of temporomandibular pain and impaired function- case report	2019	Januzzi, Eduardo and Almeida-Leite, Camila and Cunha, Thays and Fonseca, Roberta and Gonçalvez, Rafael and Oliveira, Pedro and Grossman, Eduardo and Almeida, Andre	"M.C.": "Excluded", "K.C.": "Excluded", Exclusion reason: wrong publication type	Not applicable
Arthrocentesis associated with viscosupplementation in a case of anterior disc displacement and attachment in temporomandibular joint	2019	Januzzi, Eduardo and Grossmann, Eduardo and Cunha, Thays and Goncalves, Rafael and Almeida-Leite, Camila and Ferreira, Luciano and Carvalho, Paulo and Oliveira, Pedro and Almeida, Andre	"M.C.": "Excluded", "K.C.": "Excluded", Exclusion reason: wrong drug	Not applicable
Estudio comparativo entre tratamiento con infiltraciones intraarticulares de betametasona, hialuronato de sodio y plasma rico en plaquetas en pacientes con artralgia de la articulación temporomandibular	2020	Macedo de Sousa, Bruno and López-Valverde Centeno, Antonio	"M.C.": "Excluded", "K.C.": "Excluded", Exclusion reason: foreign language	Not applicable
Relato de caso de desordem temporomandibular articular refratária: a importância da abordagem multidisciplinar ; Case report of refractory temporomandibular joint disorder: the importance of a multidisciplinary approach ; Caso clínico de trastorno de la articulación temporomandibular refractaria: la importancia de un abordaje multidisciplinario	2020	Santos, Pablo Leal Teixeira and Lima, Fern and a Ferruzzi and Mazzetto, Marcelo Oliveira and Melchior, Melissa de Oliveira and Magri, Lais Valencise and Mori, Aline Akemi	"M.C.": "Excluded", "K.C.": "Excluded", Exclusion reason: foreign language	Not applicable
Interventions for managing temporomandibular joint osteoarthritis	2012	Lovato da Silva, Claudia H and Nasser, Mona and Fedorowicz, Zbys and Al-Muharraqi, Mohammed A and de Souza, Raphael F	"M.C.": "Excluded", "K.C.": "Excluded", Exclusion reason: wrong publication type	Not applicable
Evaluation of the efficacy of the arthrocentesis associated with viscosupplementation in the treatment of the temporomandibular joint disorders: case report ; Avaliação da eficácia da artrocentese associada à viscossuplementação no tratamento das desordens temporomandibulares: relato de caso	2020	Stehling Urbano, Eduardo and Santiago, Rodrigo César and de Paula C and eia, Ana Júlia and Ferrante de Faria, Laís and de Cerqueira Luz, João Gualberto	"M.C.": "Excluded", "K.C.": "Excluded", Exclusion reason: foreign language	Not applicable
El ácido hialurónico y sus propiedades como adyuvantes para la terapia ortodóncica	2021	Vélez Trujillo, Natalia Eugenia and Celis Londoño, Melissa and Vélez Trujillo, Natalia E.	"M.C.": "Excluded", "K.C.": "Excluded", Exclusion reason: foreign language	Not applicable
A Viscossuplementação com Ácido Hialurónico no Tratamento da Disfunção Temporomandibular.	2020	Carvalho, Catarina da Costa and BRAZ, MARIA DO PRANTO VALENTE and ARAÚJO, TIAGO CAMPOS XAVIER DE	"M.C.": "Excluded", "K.C.": "Excluded", Exclusion reason: foreign language	Not applicable

Sequential infiltration of sodium hyaluronate in the temporomandibular joint with different molecular weights. Case report		Eduardo Grossmann and Roberta Fonseca and Camila Almeida-Leite and Rafael Tardin Gonçalves and Pedro Gonçalves de Oliveira and Eduardo Januzzi	"M.C.": "Excluded", "K.C.": "Excluded", Exclusion reason: wrong publication type	Not applicable
Prevalencia de los trastornos temporomandibulares en las historias clínicas de la clínica del adulto V del año 2017 al 2019 en la Universidad Santo Tomás de Bucaramanga	2021	Leal Jaimes, Camila Natalia and Guerrero Duarte, Juliana Marcela and Vergara Balaguera, Laura Jimena and Aristizábal Pérez, Jennifer and Toloza Ramírez, Emelina and Universidad Santo Tomás	"M.C.": "Excluded", "K.C.": "Excluded", Exclusion reason: foreign language	Not applicable
The Effect of Intra-Articular Injection of Hyaluronic Acid on the Degenerative Pathology of the Temporo-Mandibular Joint	2017	D, Batifol and P, Finiels and P, Jammet	"M.C.": "Included", "K.C.": "Included"	"M.C.": "Included", "K.C.": "Included"
Efficacy of arthrocentesis with injection of hyaluronic acid in the treatment of inflammatory-degenerative disease of temporomandibular joint	2021	Enas A Abdulmaged and Thair Abdul Lateef	"M.C.": "Excluded", "K.C.": "Excluded", Exclusion reason: wrong drug	Not applicable
Ultrasonography-guided invasive procedures of the temporomandibular joint	2021	Orhan, Kaan and Rozylo-Kalinowska, Ingrid	"M.C.": "Excluded", "K.C.": "Included"	"M.C.": "Excluded", "K.C.": "Excluded", Exclusion reason: wrong publication type
Evidência científica sobre o uso de ácido hialurónico no tratamento da disfunção temporo-mandibular	2017	Ferreira, Ana Lúcia Neves and Dias, Maria Carlos Real	"M.C.": "Excluded", "K.C.": "Excluded", Exclusion reason: foreign language	Not applicable
Temporomandibular eklem internal düzensizliklerinin tedavisinde sodyum hyalüronat enjeksiyonu ile birlikte yapılan artrosentez uygulaması ve konservatif tedavinin etkinliklerinin kıyaslanması ; Comparison of the efficacy of arthrocentesis with sodium hyaluronate injection and conservative treatment of temporomandibular joint internal derangements	2007	Küçük, Korhan and Ataoğlu, Hanife and Selçuk Üniversitesi, Sağlık Bilimleri Enstitüsü	"M.C.": "Excluded", "K.C.": "Excluded", Exclusion reason: foreign language	Not applicable
Evaluation of current treatment modalities for Management of Degenerative Temporomandibular joint diseases	2020	Awad Mansour, Heba Mahmoud	"M.C.": "Excluded", "K.C.": "Excluded", Exclusion reason: wrong publication type	Not applicable
Efficacy of arthrocentesis with injection of hyaluronic acid in the treatment of internal derangement of temporomandibular joint	2015	Adil Al Kayat and Thair Abdul Lateef and Enas A Abdulmajed	"M.C.": "Excluded", "K.C.": "Excluded", Exclusion reason: wrong drug	Not applicable
Eficacia del hialuronato sódico como complemento de la artroscopia en la disfunción de la articulación temporomandibular	2014	Morey Mas, Miguel Ángel and Universitat Autònoma de Barcelona. Departament de Cirurgia and Raspall, Guillermo, and Gonzalez Lagunas, Javier	"M.C.": "Excluded", "K.C.": "Excluded", Exclusion reason: foreign language	Not applicable
Hijaluronska kiselina u dentalnoj medicini ; Use of hyaluronic acid in dentistry	2021	Domislović, Petra and Šutej, Ivana	"M.C.": "Excluded", "K.C.": "Excluded", Exclusion reason: foreign language	Not applicable
Semptomatik temporomandibular eklem internal düzensizliklerinde artrosentez ve artrosentez + hyalüronik asit uygulamasının temporomandibular eklem sinoviyal sıvısındaki önemli proenflamatuvar sitokin seviyeleri üzerine etkilerinin değerlendirilmesi	2012	Özdamar, Saime Serap Moroğlu and Gürsoy, Bahar and Ağız Diş Çene Hastalıkları ve Cerrahisi Anabilim Dalı	"M.C.": "Excluded", "K.C.": "Excluded", Exclusion reason: foreign language	Not applicable
Efficacy of Arthrocentesis with Injection of Hyaluronic Acid in the Treatment of Internal Derangement of Temporomandibular Joint	2010	Shakya, Pramila and Rahman, Quazi Billur and Hossain, Shakhawat and Akhter, Mahmuda and Uddin, Md Wares	"M.C.": "Excluded", "K.C.": "Excluded", Exclusion reason: wrong drug	Not applicable
Platelet-Rich Plasma in Temporomandibular Joint Osteoarthritis Therapy: A 3-Month Follow-Up Pilot Study	2013	Machoň, V. and Řehořová, M. and Šedý, J. (Jiří) and Foltán, R.	"M.C.": "Included", "K.C.": "Excluded"	"M.C.": "Excluded", "K.C.": "Excluded", Exclusion reason: wrong intervention
Valutazione clinica e radiologica degli effetti dell’artrocentesi nei disordini dell’articolazione temporo-mandibolare	2014	Stimolo, Mirella and De Riu, Giacomo	"M.C.": "Excluded", "K.C.": "Excluded", Exclusion reason: foreign language	Not applicable

Az állkapocsízületi károsodás kezelése szteroiddal, illetve hialuronsavval = Intra-articular steroid and hyaluronic acid treatment of internal derangement of the temporomandibular joint	2018	Vingender, Szonja and Restár, László and Csomó, Krisztián Benedek and Schmidt, Péter and Hermann, Péter and Vaszilkó, Mihály	"M.C.": "Excluded", "K.C.": "Excluded", Exclusion reason: foreign language	Not applicable
Comparison of clinical outcomes of patients with nonreducing disc displacement treated by pumping and injection of sodium hyaluronate into the temporomandibular joint with or without mouth opening exercise	2005	Sato, S. and Yamaguchi, K. and Kawamura, K.	"M.C.": "Excluded", "K.C.": "Included"	"M.C.": "Excluded", "K.C.": "Excluded", Exclusion reason: wrong publication type
Artrosentez Vertigo Tedavisinde Bir Seçenek Olabilir mi?: Olgu Sunumu ; Is Arthrocentesis a Choice for Treatment of Vertigo: A Case Report	2017	KOCA, Cansu Gül and KOÇER, Gülperi and BÜLTE, Mert	"M.C.": "Excluded", "K.C.": "Excluded", Exclusion reason: foreign language	Not applicable
Tissue engineering: state of the art in oral rehabilitation	2009	Scheller, Erica L. and Krebsbach, Paul H. and Kohn, David H. and * Department of Biologic and Materials Sciences, School of Dentistry, University of Michigan, Ann Arbor, MI and Department of Biomedical Engineering, College of Engineering, University of Michigan, Ann Arbor, MI, USA	"M.C.": "Excluded", "K.C.": "Excluded", Exclusion reason: wrong publication type	Not applicable
A mastication mechanism designed for testing temporomandibular joint implants	2012	Ryan J. Frayne and Marvin Schwartz and James P. Dickey	"M.C.": "Excluded", "K.C.": "Excluded", Exclusion reason: wrong population	Not applicable
Heparin-Based Nanoparticles: An Overview of Their Applications	2018	Maria del Pilar Rodriguez-Torres and Laura Susana Acosta-Torres and Luis Arm and o Diaz-Torres and Faheem Ahmed	"M.C.": "Excluded", "K.C.": "Excluded", Exclusion reason: wrong population	Not applicable
